# Evaluating the acute flaccid paralysis surveillance system in South Africa, 2005-2009 - an analysis of secondary data

**DOI:** 10.11604/pamj.2013.14.86.2032

**Published:** 2013-03-05

**Authors:** Landiwe Siphumelele Khuzwayo, Lazarus Rugare Kuonza, Ntombenhle Judith Ngcobo

**Affiliations:** 1 South African Field Epidemiology and Laboratory Training Programme, National Institute for Communicable Diseases, Johannesburg, South Africa; 2 School of Health Systems and Public Health, Faculty of Health Sciences, University of Pretoria, South Africa; 3 Expanded Programme on Immunization, National Department of Health, Pretoria, South Africa

**Keywords:** South Africa, acute flaccid paralysis, surveillance, evaluation, poliovirus

## Abstract

**Introduction:**

Acute Flaccid Paralysis (AFP) surveillance was adopted by World Health Organization (WHO) to monitor progress towards poliomyelitis eradication. South Africa Department of Health (DoH) routinely collects AFP surveillance data but has no documented evidence of its epidemiological use. The study discusses the epidemiology of AFP in South Africa from 2005-9, evaluates performance of the AFP surveillance system, and identifies components that require strengthening.

**Methods:**

A retrospective descriptive analysis was conducted on secondary AFP surveillance data for South Africa for the period 2005-2009, consisting of all children.

**Results:**

South Africa reported 1501 AFP cases between 2005 and 2009. Of these, 67.2% were <5years of age, and 54.3% were male. None of the cases were confirmed poliomyelitis, and ten (0.7%) were classified as polio-compatible. The national annualized non-polio AFP detection rate increased from 1.6 in 2005 to 2.1 non-polio AFP cases/100,000 children <15years in 2008-9. All performance indicators met the WHO-specified targets except two. Between 2007 and 2009, 51.5%, 55.3% and 65% of specimens, respectively, reached the laboratory within 72hours of being sent (WHO target is ≥80%). Proportion of stool specimens where non-polio enterovirus was isolated decreased from 22.5% in 2006 to <1% in 2008 and 2009 (WHO target is ≥10%).

**Conclusion:**

The AFP surveillance system met most WHO-specified epidemiological and laboratory performance standards. The surveillance programme needs to address problems of delayed specimen arrival to the laboratory and incomplete documentation of laboratory findings in the national AFP surveillance database.

## Introduction

Poliomyelitis is a highly contagious viral disease caused by infection with the poliovirus (serotypes 1, 2 and 3) [1]. The poliovirus infects mostly children below the age of five years, and in up to 1% of those infected the virus invades the central nervous system leading to muscle weakness and irreversible paralysis (usually in the lower limbs), often progressing to breathing problems, and death [1, 2] In 1988, the forty-first World Health Assembly (WHA) resolved to eradicate poliomyelitis worldwide by the year 2000. The resolution marked the launch of the Global Polio Eradication Initiative (GPEI), spearheaded by the World Health Organization (WHO), and member states (including South Africa) adopted a number of strategies to ensure the success of the initiative [2, 3]. Although the goal has not been achieved, the GPEI has made significant progress, reducing the global incidence of poliomyelitis by more than 99% from an estimated 350,000 cases in 1988 to 1604 cases reported in 2009. The number of polio-endemic countries has also decreased from 125 countries in 1988, to four countries (Nigeria, Pakistan, India and Afghanistan) by 2010 [4].

Acute Flaccid Paralysis (AFP) is a clinical syndrome characterized by a sudden onset of weakness of a limb, described as flaccid (reduced tone) in a child below 15 years of age [5]. AFP mimics the clinical presentation of poliomyelitis, hence AFP surveillance was adopted globally as a key strategy for monitoring the progress of the polio eradication initiative [5–7]. A good AFP surveillance system serves as a sensitive instrument for detecting potential poliomyelitis cases and thus alerting health managers and clinicians to timely institute appropriate interventions to interrupt any poliovirus transmission. Effective AFP surveillance is also crucial for verifying, with confidence, the absence of wild poliovirus circulation in countries that are no longer reporting cases of poliomyelitis. [2, 8].

In South Africa the last case of poliomyelitis due to the wild poliovirus was reported in 1989, and the country was awarded a polio free status in 2006, certifying that the wild poliovirus is no longer circulating in the country [2, 9]. However the country remains at risk of wild poliovirus re-importation from the remaining polio-endemic countries. The Expanded Programme on Immunisation - South Africa (EPI-SA) has routinely collected AFP surveillance data since 1997 [9], but only limited epidemiological analysis of the data has been conducted. Regular analysis of data generated from an AFP surveillance system is important in evaluating and improving the performance of the system. This ensures optimal performance of the system and guarantees timely detection of wild poliovirus re-importation. The WHO has developed a set of performance indicators in order to ensure that AFP surveillance is adequately conducted to accurately guide the polio eradication initiative [2, 5–7]. This paper discusses the epidemiological distribution of AFP in South Africa between 2005 and 2009, evaluates the performance of the AFP surveillance system, and identifies components that require strengthening.

## Methods

### Study setting and design

South Africa is located at the southern tip of Africa and borders with Namibia, Botswana and Zimbabwe to the north, Mozambique and Swaziland to the north-east, and has Lesotho is an independent country wholly surrounded by South African territory. The country is divided into nine provinces, with an estimated total population of about 50 million people (according to 2010 estimates). The population of children under the age of 15 years is estimated to be about 15.4 million.

A retrospective descriptive study was conducted using secondary AFP surveillance data routinely collected between January 2005 and December 2009 by the EPI-SA of the South Africa National Department of Health. All AFP cases reported to the EPI - SA during this period, from all the nine provinces, were included in the study.

### The AFP surveillance system in South Africa

In South Africa an AFP case is defined as any child below the age of 15years who develops acute onset of focal weakness or paralysis characterized as flaccid (including Guillain Barre Syndrome), without any other obvious cause [6, 7]. When a patient meeting the AFP case definition is seen at a health facility the health care practitioners conduct comprehensive investigations to rule out poliovirus as a cause of the paralysis. The investigation involves taking a detailed history, conducting a systematic examination, and collecting two stool specimens, 24 to 48 hours apart, within 14days of onset of symptoms. The specimens are sent to the WHO accredited poliovirus isolation laboratory at the National Institute for Communicable Disease (NICD) for enterovirus analysis. A case investigation form is then completed and sent through the levels of the health system to the EPI-SA. The case investigation report includes demographic information, clinical history, immunisation history, adequacy of stool specimen collection and information about the 60 day follow up examination.

An AFP case where two adequate stool specimens are submitted for analysis and no poliovirus is isolated is classified as a non-polio case (and is said to have been discarded). A case where the stool specimens are deemed inadequate but has no residual paralysis after 60 days of onset of symptoms is also classified as a non-polio case (discarded). A case that has inadequate stool specimens and has residual paralysis after 60 days, is lost to follow up or dies within 60 days of symptom onset is referred to the National Polio Expert Committee (NPEC) for a detailed review and final classification (i.e. for a decision on whether the case is compatible with polio or should be discarded). All cases that are reported and later found to be incompatible with the AFP case definition are de-notified (deleted from the database).

### AFP surveillance indicators

The WHO has set some minimum performance standards that should be used to evaluate the quality of AFP surveillance. In this study, the performance of the AFP surveillance system was evaluated using the following WHO-specified indicators [2, 5–7]:

1. *Annualized non-polio AFP rate:* This is an indicator of the sensitivity of the AFP surveillance system. The system should be able to detect at least two AFP cases per 100,000 children below the age of 15 years. The rate is based on the fact that in the absence of wild poliovirus, cases of AFP continue to occur due to other causes like Guillain-Barré syndrome, transverse myelitis, etc
2. *Stool adequacy:* Adequate stools are defined as two stool specimens collected from an AFP patient 24-48hours apart and within 14 days of onset of symptoms. At least 80% of all AFP cases should have adequate stool specimens.
3. *Condition of stool on arrival at laboratory:* At least 80% of the stool specimens should arrive at the WHO accredited laboratory in “good condition”. A stool specimen is said to have arrived in good condition if it was transported under reverse cold chain conditions (with ice packs and a temperature indicator) and was received by the WHO accredited polio isolation laboratory in sufficient quantity (at least 8grams) and with correct documentation
4. *Timeliness of case investigation:*At least 80% of AFP cases should be investigated within 48 hours of being notified.
5. *Timeliness of transportation of specimens to the laboratory:* At least 80% of stool specimens collected from AFP cases should arrive at a WHO accredited polio isolation laboratory within 72 hours of being sent.
6. *Timeliness of specimen processing in the laboratory:* At least 80% of specimen results should be sent from the polio isolation laboratory within 28 days of specimen receipt by the laboratory
7. *Non-polio enterovirus isolation rate:* At least 10% of stool specimens submitted to the laboratory should have non-polio enterovirus isolated. This is an indicator of the quality of the reverse cold chain and how well the laboratory is able to perform in the routine isolation of enterovirus.
8. *60-day follow up examination:* At least 80% of AFP cases requiring a follow-up examination should be examined at 60 days after the onset of paralysis, to verify the presence of residual paralysis or weakness.

### Data analysis

Data were analyzed using the Epi Info statistical software (version 3.5.1; Centers for Disease Control and Prevention, Atlanta, United States). Descriptive analyses were conducted to describe the epidemiology of AFP in South Africa and to generate statistics based on the standard WHO-specified performance indicators for AFP surveillance.

### Ethical considerations

All the data analyzed in the study were captured from the National Department of Health’s EPI - SA data base, no further information was obtained from patients. Ethical clearance to conduct the study was granted by the Faculty of Health Sciences Research Ethics Committee of the University of Pretoria (Protocol number S17/2011) and further written permission was granted by the director general of the National Department of Health.

## Results

Cumulatively 1616 AFP cases were reported to the EPI-SA between January 2005 and December 2009. Out of these, 115 records were excluded from the analysis (96 had been de-notified after being classified as not AFP by the NPEC, 15 were duplicate records, three cases had onset of paralysis before January 2005 and one record had no data).

Among the 1501 AFP cases analyzed, 67.2% were below 5 years of age, and 54.3% were male. Almost half (45.2%) of the cases had unknown polio immunization status, and of the 823 that had known immunization status, 379 (46.1%) were lagging behind with the immunization schedule (Table 1).

**Table 1:** Background characteristics of AFP cases reported in South Africa between January 2005 and December 2009

Characteristic	Frequency	
	(n = 1501)	%
Sex		
Male	815	54.3
Female	686	45.7
Age-group categories		
<1 years	63	4.2
1-5 years	946	63.0
6-10 years	335	22.3
11-15 years	157	10.5
Clinical symptoms		
Fever at onset	692	39.4
Asymmetric paralysis	508	33.8
Paralysis progressed > 3 days	872	58.1
**Polio immunization status**		
Partially immunized and on schedule	379	25.2
Partially immunized and not on schedule	444	29.6
Unknown immunization status	678	45.2

**Figure 1:**
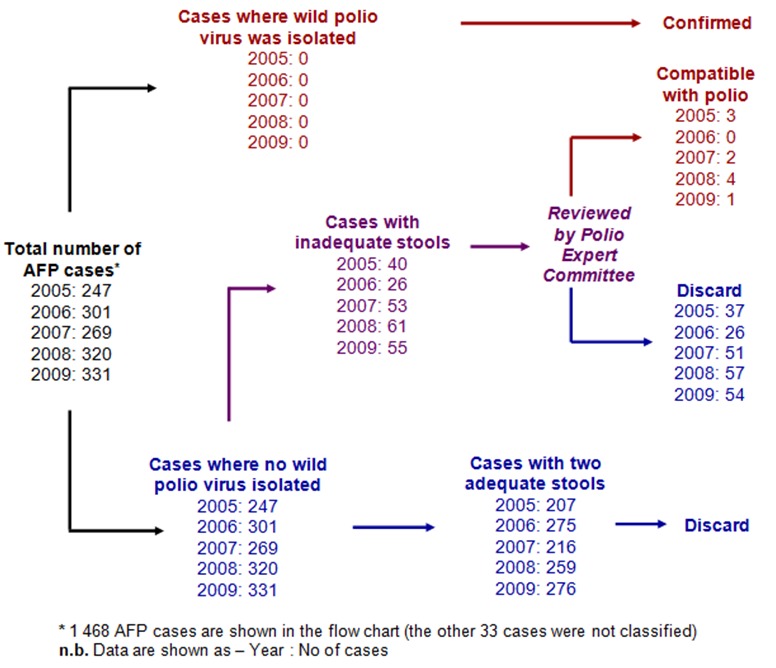
Flow chart showing the virological classification of AFP cases reported in South Africa between 2005 and 2009

The AFP cases were classified according to the WHO virological classification flowchart (Figure 1). None of the cases were classified as poliomyelitis, and the National Polio Expert Committee classified ten cases (0.7%) as polio-compatible (Figure 1). A variety of diagnoses were identified as the causes of the AFP, though 42% of the cases had no explicit diagnoses captured (other than that of AFP). Among the 751 cases that had definite diagnoses, the most common were Guillain-Barré Syndrome, 321 (42.7%), and meningitis, 117 (15.6%) (Table 2).

**Table 2:** Final diagnoses given for AFP cases reported in South Africa between 2005 and 2009

Diagnosis	Frequency	
	n	%
**Acute Flaccid Paralysis**	630	42.0
**Gullian-Barre Syndrome**	321	21.4
**Meningitis**	117	7.8
**Encephalitis**	26	1.7
**Organophosphate poisoning**	22	1.5
**Gastro Enteritis**	20	1.3
**Hemiparesis**	19	1.3
**Hemiplegia**	19	1.3
**Others**	207	13.8
**No Diagnosis**	120	8.0
**Total**	1501	100.00

^*^ N.b. the category “others” include:cerebella disease, cerebral lesion, dehydration, delayed milestone, drug overdose, etc

**Figure 2:**
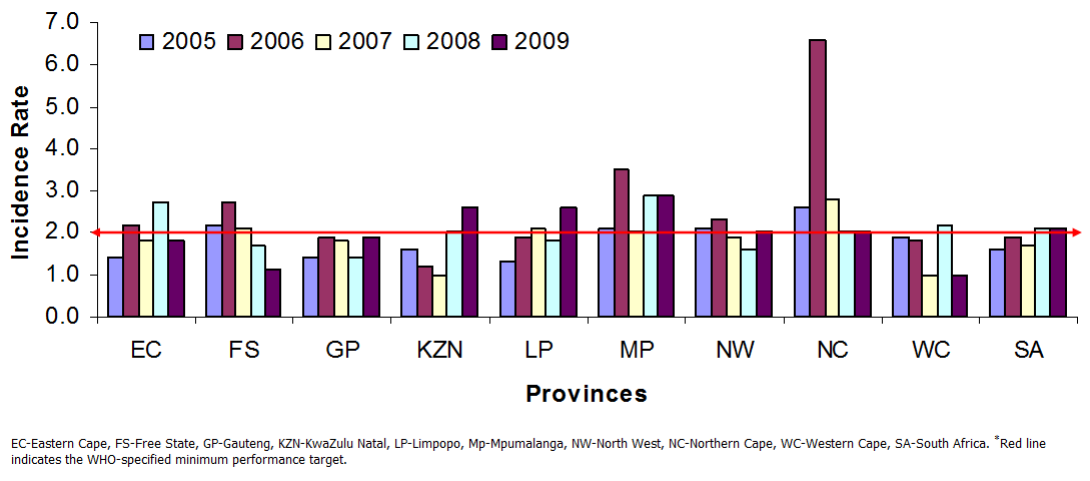
Annualized non-polio AFP rates by year for each province in South Africa, 2005-2009

Cumulatively the South Africa's annualized non-polio AFP detection rate was 1.8 AFP cases per 100 000 population below 15 years per year between 2005 and 2009. The country's AFP detection rate increased from 1.6 cases in 2005 to 2.1 cases per 100 000 population below 15 years in 2009 (Table 3). Disaggregating the national cumulative AFP rates by province shows that the majority of the provinces (except Mpumalanga and Northern Cape), consistently failed to surpass the WHO minimum target of 2 AFP cases per 100,000 population under 15years during the five year period (Figure 2). However some provinces (Eastern Cape, KwaZulu Natal and Limpopo) showed improvements in the AFP rates between 2005 and 2009.

**Table 3:** AFP surveillance performance indicators for South Africa, 2005-2009

Performance indicator	Target	Country performance				
		2005	2006	2007	2008	2009
Annualized non-polio AFP rate /100,000 <15 yrs population	≥2	1.6	1.9	1.7	2.1	2.1
Proportion of AFP cases investigated (out of the total notified)	≥80%	100%	100%	100%	100%	100%
Proportion of AFP cases investigated within 48 hours of notification	≥80%	81.1%	79.6%	83.2%	81.6%	78.8%
Proportion of AFP cases followed up at 60 days of onset of symptoms	≥80%	100%	100%	100%	100%	100%
Proportion of AFP cases with two adequate stool specimens^†^	≥80%	79.9%	85.5%	82.6%	81.6%	84.6%
Proportion of specimens that arrived at a WHO accredited laboratory <3 days of being sent*	≥80%	–	–	51.9%	55.3%	65.0%
Proportion of stool specimens arriving at the laboratory in good condition	≥80%	96.4%	99.0%	97.7%	94.6%	98.1%
Proportion of stool specimens from which non-polio enterovirus was isolated	≥10%	13.2%	22.5%	12.9%	0%	0.6%
Proportion of stool specimens with results sent from laboratory <28 days of receipt by laboratory	≥80%	94.1%	98.5%	96.3%	95.00%	95.8%

* For 2005 and 2006 there were dates of receipt of specimens by the laboratory (hence indicator could not be calculated)

**Figure 3:**
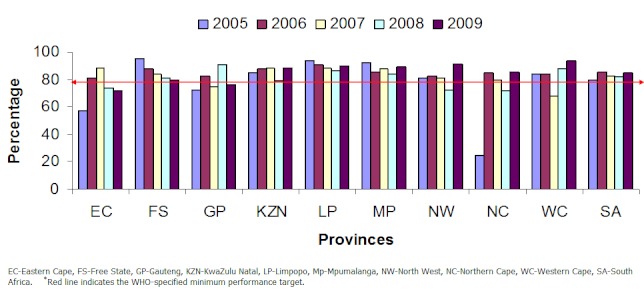
Proportion of AFP cases with adequate stool specimens per year, by province, South Africa 2005-2009

The proportion of AFP cases with two adequate stool specimens was constantly above the minimum target of ≥80%, except in 2005 where it was 79.9% (Figure 3). All AFP cases that were due for 60 day follow up were examined at 60 days of onset of symptoms (Table 3).

Records for 2005 and 2006 had no data to indicate the timeliness of arrival of stool specimens to the WHO accredited poliovirus isolation laboratory. For the period 2007 to 2009, the proportion of specimens arriving at the laboratory within three days of being sent were constantly below the WHO minimum target of at least 80% (Table 3). The proportion of stool specimens where non-polio enterovirus was isolated decreased from 22.5% in 2006 to almost nil in 2008 and 2009 (Table 3).

## Discussion

South Africa remains at risk of wild poliovirus importation from countries where the virus is still circulating. The non-polio AFP detection rate is an indicator of the sensitivity of the AFP surveillance system in a country, and illustrates the ability of a country to detect a case of poliomyelitis if wild poliovirus was to be re-imported into the country. Results from South Africa's AFP surveillance system show that the non-polio AFP detection rate increased from 1.6 in 2005 to 2.1 (cases per 100,000 population 6, 7]. Though South Africa's AFP rate was on an increasing trend between 2005 and 2009, the WHO target was only met in 2008 and 2009. The increase in the AFP rate during that period appears to be due to the change of AFP case detection targets for the region in 2006, following an outbreak of imported wild poliovirus in Angola, which later spread to neighboring countries like Namibia, DRC, Central African Republic, and Burundi [4, 10]. The WHO then increased the minimum AFP detection rates for countries in the region from 1 non-polio AFP case to 2 non-polio AFP cases per 100 000 children [11]. Furthermore, on disaggregating the non-polio AFP rates by province, some provinces seem to have performed exceptionally well (Mpumalanga and Northern Cape), while others have continuously failed to meet the new target (Gauteng and Western Cape). Hence looking at the cumulative national performance tends to mask the underperforming sub-national levels, and these areas may possibly become pockets of transmission where polio virus circulation could go undetected if the virus is re-imported into the country. Efforts should therefore be made to strengthen the sub-national AFP surveillance system.

The success of an AFP surveillance system does not only depend on the detection of AFP cases, but also hinges on the investigation and reporting of the cases. Results from South Africa's AFP surveillance programme show that, the country's overall proportion of adequate stool specimens, during the investigation of AFP cases, was maintained above the WHO-specified national target of at least 80% from 2006 through 2009. This performance was also reflected at the sub-national levels, with most of the provinces maintaining the stool adequacy rate above the target. A stool specimen is adequate if collected within 14 days of onset of symptoms, transported to the laboratory under reverse cold chain conditions (between frozen ice packs and with a temperature indicator) and received by a WHO accredited laboratory in sufficient quantity (at least 8grams), in good condition and with appropriate documentation [6, 7]. Laboratory analysis is critical to the confirmation of the poliovirus, therefore the stool specimens that are sent to the laboratory should be of sufficient quality to enable the laboratory to identify the poliovirus or to rule out its presence with a high degree of confidence. Since no poliovirus was isolated during this study period with such high stool adequacy rates, the country can be confident that there was no wild-poliovirus circulating in the country.

Ten AFP cases were classified as compatible with poliomyelitis by the National Polio Expert Review Committee. An AFP case is classified as polio compatible if the stool specimens were not adequate enough to rule out the poliovirus and the patient either had polio-compatible residual paralysis at 60 days, died within 60 days or was lost to follow up before investigations could be finalized to rule out poliomyelitis as a cause [6, 7]. Polio compatible cases are generally considered to result from failures of the AFP surveillance system; they show that the system may not be robust enough to exclude the existence of wild poliovirus circulation with certainty. A study done in India (in 2000) reported that clusters of polio compatible cases tended to occur in areas with continuing wild poliovirus transmission, suggesting that these were actually missed polio cases [12]. It is therefore important to monitor polio compatible cases for clustering by geographical area and by time [5].

Guillain Barre Syndrome was the most common cause of AFP in South Africa. This is consistent with findings from other countries [13–15]. Of concern however is that the majority of the AFP cases did not have specific diagnosis assigned to them, they were simply categorized as AFP. Acute flaccid paralysis is a clinical syndrome with a broad array of possible differential diagnoses, hence accurate diagnosis of the cause of AFP is important for guiding therapy and prognosis. AFP case investigation should always aim to isolate the reason of the paralysis and the surveillance officers should follow up all investigated cases and make certain that the final diagnoses are accurately captured on the AFP surveillance database.

When investigating an AFP case, the timeliness of transportation of the stool specimens is important, the polio enteroviruses should survive until the time of analysis for the laboratory to be able to isolate them. According to the WHO-specified national targets for AFP surveillance, at least 80% of stool specimens must arrive at the laboratory within 72 hours of collection [5]. Results from this study reveal that South Africa failed to meet this target throughout the study period, as the proportion of specimens that arrived at the laboratory within 72 hours was consistently less than 80%. The poor performance on this indicator points to flaws in the way the AFP specimens are transported. At this point it is impossible to point out with certainty, where the delays could have occurred. In South Africa, the national AFP surveillance programme has put in place a courier service which is available on request, all days of the week, to transport stool specimens from health facilities to the NICD. Hence the delays may perhaps occur at any stage of the specimen transportation, from the health facility level, within the courier services, or in the movement of specimens within the units of the laboratory network. A separate study looking at the transportation of specimens from health facilities to the NICD can unearth some of these loopholes.

Furthermore, the results show that the proportion of specimens where non-polio enterovirus was isolated decreased dramatically between 2006 and 2009, and the country failed to meet the WHO-specified target of at least 10% in 2008 and 2009. This indicator evaluates the integrity and viability of stool specimens received by the laboratory and also assesses how well the laboratory performs in the routine isolation of enteroviruses. Interestingly, in contrast to these results from the EPI (SA), data from the enterovirus isolation unit of the National Institute for Communicable Disease shows that the non-polio enterovirus isolation rates for stool specimens submitted to the laboratory for AFP investigation in 2008 and 2009 were 8.3% and 10.0% respectively [16, 17]. These non-polio enterovirus isolation rates are more in line with the WHO-specified standards and are much higher than the EPI-SA rates which were below 1%. The discrepancies suggest either a lack of dissemination of results from the NICD to the EPI-SA, or a lack of proper documentation of laboratory results on the AFP database, by the surveillance officers in the EPI-SA unit. This incongruity may perhaps also explain why almost half of the AFP cases in the data base did not have final diagnoses assigned to them; and suggests that the final outcomes of the investigations may have been made by the laboratory but were simply not recorded onto the case investigation records. There is therefore a need to strengthen the follow up of specimens submitted to the polio isolation laboratory and the proper documentation of the findings onto the relevant reports.

## Conclusion

The AFP surveillance system in South Africa met most of the WHO-specified epidemiological and laboratory performance standards. However, the evaluation points to problems in the timeliness of transportation of stool specimens from the health facilities to the national polio isolation laboratory and also shows weaknesses in the documentation of laboratory findings in the national AFP surveillance database. Stool specimen transportation from health facilities to the WHO-accredited poliovirus isolation laboratory should be critically reviewed to identify and address the loopholes causing delays in specimen arrival to the laboratory. The EPI-SA should strengthen the follow-up of stool specimens submitted to the laboratory and the documentation of the laboratory findings onto the AFP surveillance database. It is also necessary to continually strengthen the AFP surveillance system at the underperforming sub-national levels, to ensure that the system is robust enough to document the absence of the wild poliovirus in the country as well as to timely detect any poliovirus re-importation.

## References

[1] Heymann DL (2008). Control of communicable diseases manual.

[2] EPI (SA) (1998). EPI disease surveillance field guide: guidelines for detecting, reporting, investigating and responding to EPI priority diseases.

[3] WHO (2000). Forty-First World Health Assembly -Global eradication of poliomyelitis by the year. http://www.polioeradication.org/content/publications/19880513_resolution.pdf.

[4] CDC (2011). Tracking Progress Toward Global Polio Eradication Worldwide, 2009-2010. MMWR.

[5] WHO (2003). Recommended standards for surveillance of selected vaccine preventable diseases: Vaccines and Biologicals.

[6] WHO (1998). Acute flaccid paralysis surveillance: the surveillance strategy for poliomyelitis eradication. Wkly Epidemiol Rec.

[7] WHO (1996). Field Guide: For Supplementary Activities aimed at achieving polio eradication-1996 revision.

[8] Heymann DL (2004). Polio eradication: finishing the job and protecting the investment. Bull World Health Organ..

[9] National Department of Health (2006). South Africa Declared Polio Free. http://www.doh.gov.za/docs/pr/2006/pr1020.html.

[10] CDC (2011). Progress Toward Interrupting Wild Poliovirus Circulation in Countries With Reestablished Transmission?. Africa, 2009-20 MMWR.

[11] WHO Global Polio Eradication Initiative - AFP/Polio case count summary. http://apps.who.int/immunization_monitoring/en/diseases/poliomyelitis/case_count.cfm.

[12] Kathryn A Kohler, Gary W Hlady, Kaushik Banerjee, Dhananjoy Gupta, Paul Francis, Sunita Durrani, Patrick L F Zuber, Roland W Sutter (2003). Compatible poliomyelitis cases in India during 2000. Bull World Health Organ..

[13] Arthur Marx, Jonathan D Glass, Roland W Sutter (2000). Differential diagnosis of acute flaccid paralysis and its role in poliomyelitis surveillance. Epidemiol Rev..

[14] Lam RMK, Tsang THF, Chan KY, Lau YL, Lim WL, Lam TH, Leung NK (2005). Surveillance of acute flaccid paralysis in Hong Kong: 1997 to 2002. Hong Kong Med J..

[15] D'Errico Marcello M, Barbadoro Pamela, Bacelli Sonia, Esponoto Elisabetta, Moroni Vania, Scaccia Federica, Luana Tantucci, Emelia Prospero (2008). Surveillance of acute flaccid paralysis in the Marches Region (Italy) 1997-2007. BMC Infectious Diseases.

[16] Jo McAnerney, Nicksy Gumede-Moeletsi, Peter Coetzee, Alfred Mawela, Olivia Lentsoane, Shelina Moonsamy, Busisiwe Guliwe, Cheryl Cohen, Adrian Puren (2009). Acute flaccid paralysis (afp) surveillance, 2008. Communicable Diseases Surveillance Bulletin.

[17] Cheryl Cohen, Nicksy Gumede-Moeletsi, Jo McAnerney, Shelina Moonsamy, Adrian Puren (2010). Acute flaccid paralysis surveillance, 2009. Communicable Diseases Surveillance Bulletin.

